# Acute Endovascular Reperfusion Therapy in Ischemic Stroke: A Systematic Review and Meta-Analysis of Randomized Controlled Trials

**DOI:** 10.1371/journal.pone.0122806

**Published:** 2015-04-27

**Authors:** Toshiya Osanai, Vinay Pasupuleti, Abhishek Deshpande, Priyaleela Thota, Yuani Roman, Adrian V. Hernandez, Ken Uchino

**Affiliations:** 1 Cerebrovascular Center, Cleveland Clinic, Cleveland, Ohio, United States of America; 2 Department of Medicine, Case Western Reserve University, Cleveland, Ohio, United States of America; 3 Medicine Institute Center for Value Based Care Research, Cleveland Clinic, Cleveland, Ohio, United States of America; 4 Unidad de Análisis y Generación de Evidencias en Salud Pública (UNAGESP), Instituto Nacional de Salud, Lima, Peru; 5 Medical School, Universidad Peruana de Ciencias Aplicadas (UPC), Lima, Peru; 6 Health Outcomes and Clinical Epidemiology Section, Department of Quantitative Health Sciences, Lerner Research Institute, Cleveland Clinic, Cleveland, Ohio, United States of America; University of Münster, GERMANY

## Abstract

**Background:**

Randomized controlled trials (RCTs) of endovascular therapy for acute ischemic stroke have had inconsistent results. We evaluated the efficacy and safety of endovascular therapy in published RCTs.

**Methods:**

We performed a systematic review of RCTs of endovascular therapy with thrombolytic or mechanical reperfusion compared with interventions without endovascular therapy. Primary outcome was the frequency of good functional outcome (modified Rankin scale (mRS) of 0-2 at 90 days) and secondary outcomes were mortality at 90 days and symptomatic intracranial hemorrhage (sICH). Random-effects meta-analysis was performed and the Cochrane risk of bias assessment was used to evaluate quality of evidence.

**Results:**

Ten studies involving 1,612 subjects were included. Endovascular therapy was not significantly associated with good functional outcome (Relative Risk [RR] =1.17; 95% CI, 0.97 to 1.42; p=0.10 and Absolute Risk Difference [ARD] =7%; 95%CI -0.1% to 14%; p=0.05); heterogeneity was moderate among studies (I^2^=30%). Mortality was unchanged with endovascular therapy (RR=0.92; 95 % CI, 0.75 to 1.13; p=0.45) and there was no difference in sICH (RR=1.20; 95 % CI, 0.79 to 1.82; p=0.39). The quality of evidence was low for all outcomes and the recommendation is weak for the use of endovascular therapy as per GRADE methodology.

**Conclusions:**

Intra-arterial therapy did not show significant increase in good outcomes and no changes in either mortality or sICH in patients with acute ischemic stroke. We need further RCTs with better design and quality to evaluate the true efficacy of endovascular therapy.

## Introduction

Acute endovascular reperfusion is becoming an important part of acute ischemic stroke therapy, but randomized controlled trials (RCTs) have had inconsistent results. Prolyse in Acute Cerebral Thromboembolism 2 trial (PROACT-2), showed that the intra-arterial (IA) thrombolysis with pro-urokinase for middle cerebral artery (MCA) occlusion increased the likelihood of good outcome defined by modified Rankin scale (mRS) 2 or less.[1] A subsequent study of IA thrombolysis, MELT Japan, was underpowered as only were analyzed 114 patients out of the 200 planned. This study was aborted because of approval of intravenous infusion of recombinant tissue plasminogen activator in Japan and the primary outcome, the proportion of patients with favorable outcomes (mRS scores of 0 to 2) at 90 days did not reach statistical significance.[2] Since 2004 several mechanical thrombectomy devices have been approved by government regulatory authorities according to the results of the non-randomized studies.[3–7]. These uncontrolled studies have reported higher likelihood of good outcomes among those who achieved good recanalization compared to those in whom the arterial occlusion could not be opened. In 2013, three RCTs have been published to test the efficacy of mechanical thrombectomy.[8–10]

Three prior systematic reviews of RCTs focused on the question of IA thrombolysis compared to placebo or intravenous (IV) thrombolysis. However these analyses did not include a large number of subjects with mechanical thrombectomy approach.[11–13] With the recent publication of the three RCTs using mechanical thrombectomy, we performed a systematic review and meta-analysis of RCTs comparing endovascular treatment for acute ischemic stroke with control treatment.

## Methods

### Data sources and searches

A comprehensive literature search using PubMed-Medline, The Cochrane library, The Web of Science, and Scopus from database inception through July 24, 2013 was conducted by three investigators (OT, VP and AD). The following keywords were used: acute ischemic stroke, endovascular therapy, intra-arterial therapy, catheter-based therapy, Merci, Penumbra, Solitaire, Trevo, stent, GpIIb/IIIa antagonist, and randomized controlled trial. The search strategy of PubMed is available as Appendix A in S1 File.

### Study selection and data extraction

The following predetermined inclusion criteria were used: (i) RCTs, (ii) studies evaluating the efficacy of endovascular treatments for acute ischemic stroke in comparison with a control group without endovascular treatment (placebo, intravenous therapy, standard care [i.e. usual clinical practice at the time of the trial]); (iii) study in any language. Our exclusion criteria were: (i) no control group; (ii) outcome measures data were not available or could not be extracted from the study groups. A list of retrieved articles was reviewed independently by 3 investigators (OT, VP and AD) in order to choose potentially relevant articles, and disagreements about particular studies were discussed and resolved by consensus.

Two reviewers (OT and VP) independently extracted data from studies. The following information was extracted: age, study years, geographic location, study phase, blinding, and requirement of arterial occlusion for randomization, time to randomization, time to endovascular therapy, allocated therapy, and National Institutes of Health Stroke Scale (NIHSS) score at baseline. Outcome data of interest were mRS at 90 days, mortality, and symptomatic ICH (sICH). We defined good functional outcome as mRS between 0 and 2 points. One other author (AVH) reviewed the extractions for inconsistencies, and the three investigators (AVH, OT and VP) reached consensus.

### The Cochrane risk of bias evaluation

The risk of bias for each study was evaluated using the Cochrane Collaboration tool for assessing risk of bias in randomized controlled trials. [14] The following items were evaluated:

- Generation of the allocation sequence (selection bias)
- Concealment of the allocation sequence (selection bias)
- Blinding (detection and performance bias), blinding of participants and personnel and blinding of outcome assessment
- Incomplete outcome data (attrition bias)
- Selective outcome reporting (reporting bias)
- Other biases

For each randomized controlled trial each item was described as: low risk of bias, high risk of bias and unclear risk of bias.[14] As secondary source of evaluation of quality of evidence, we also used the Grading of Recommendations Assessment, Development and Evaluation (GRADE) approach per randomized controlled trial (Appendix B in S1 File). One other author (AVH) reviewed the evaluations for inconsistencies, and the three investigators (AVH, OT and VP) reached consensus.

### Data synthesis and analysis

Our systematic review and meta-analysis followed the Preferred Reporting Items for Systematic Reviews and Meta-Analyses (PRISMA) statement (Table A in S1 File).[15] We used the Mantel-Haenszel (MH) method to calculate pooled Relative risks (RRs) and Absolute Risk Differences (ARD) and their 95% CIs. Statistical heterogeneity was evaluated with the Cochran χ^2^ and the I^2^ statistics. I^2^ values of 30–60% represented a moderate level of heterogeneity. A P value of < 0.1 for χ^2^ was defined as indicating the presence of heterogeneity. Some degree of heterogeneity was expected and therefore to take into account sources of heterogeneity, several subgroup meta-analyses were pre-specified: (i) type of endovascular therapy (IA thrombolysis or mechanical thrombectomy), (ii) type of comparator (IV thrombolysis or no requirement for IV thrombolysis), and (iii) vessel occlusion status (required demonstrated vessel occlusion for randomization or not). We also used cumulative meta-analysis to show the evolution of risks over time. DerSimonian and Laird random effects models were used for meta-analyses.[16] To examine bias in the results of the meta-analyses, the Egger’s test was used to evaluate asymmetry of the funnel plots. We used Review Manager (RevMan 5.0, Oxford, UK; The Cochrane Collaboration, 2008) and R metafor software (www.metafor-project.org).

## Results

### Eligible studies

**Fig 1:**
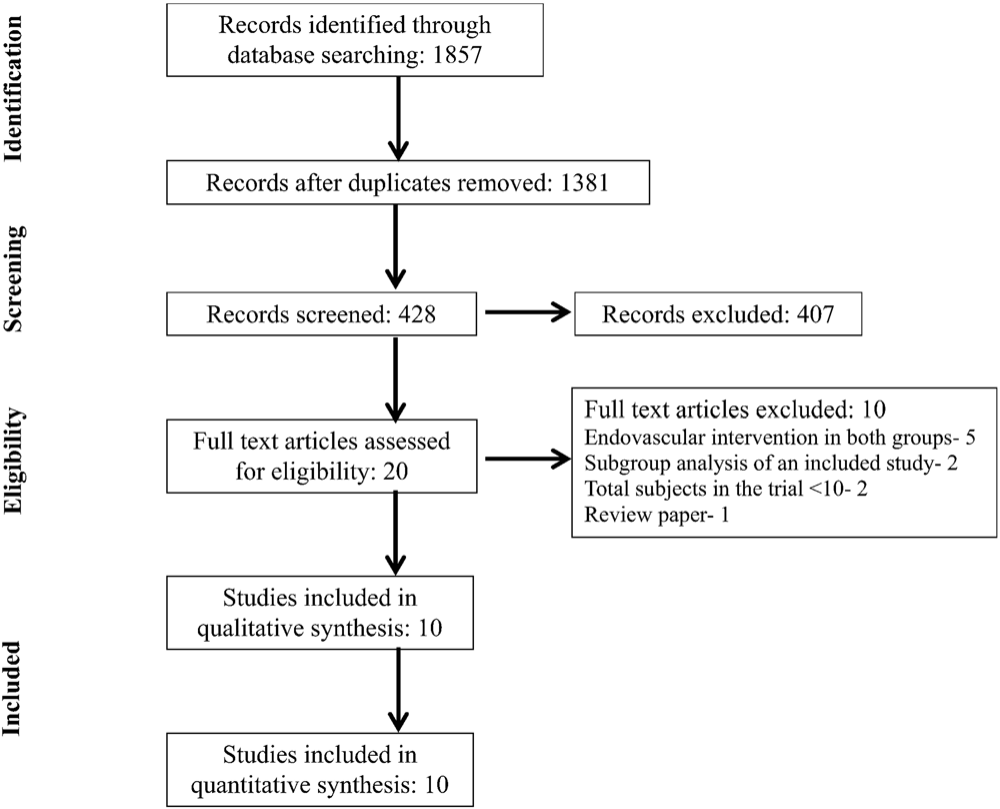
Flow diagram of selected studies.

Our search identified 1857 publications. After removing duplicates and screening titles of the studies, 428 articles were selected based on relevance to the study topic. After screening the abstracts of these potentially relevant articles, 20 were selected for full-text review based on relevance to the study topic (Fig 1). Ten RCTs involving 1,612 subjects that reported outcomes data of endovascular therapy in acute ischemic stroke in comparison to a control (no endovascular therapy) were included in the systematic review and meta-analyses. The reasons for exclusion of the remaining 10 articles are listed in Fig 1. Reference lists of all included studies were also searched and no relevant articles were identified for inclusion.

### Study characteristics

Table 1 summarizes the main characteristics of the included studies. Of the 10 trials included, 7 trials[1,2,17–21] had IA thrombolysis only in the active treatment arms, while 3 trials[8–10] allowed mechanical thrombectomy devices in the active treatment arms. Five trials required that vessel occlusion was necessary for inclusion of patients (3 studies of MCA occlusions[1,2,8,18], 1 of anterior circulation occlusions including MCAs [10,20], 1 limited to cerebral vessel occlusion located in posterior circulation.[21] Five trials did not require the cerebral vessel occlusion [9,17,19]. Outcomes were determined by using mRS 0–2 at 90 days in 8 studies. Remaining 2 studies used mRS 0–1 and 0–3 as primary outcomes and did not report the distribution between groups. For one study mRS 0–2 was reported in a secondary publication[18]. Thus we excluded one study reporting primary outcome as mRS 0–3, when we evaluate good functional outcome. Various mechanical devices were used in the studies which compared mechanical thrombectomy with control treatment. Broderick *et al*[8] allowed the use of Merci retrieval, Penumbra system or Solitaire FR. Kidwell *et al*[10] permitted the treatment with Merci retrieval or Penumbra. Ciccone *et al* [9] did not reveal the brand of mechanical thrombectomy device. A total of 1,612 patients were included in the meta-analysis with sample sizes ranging from 16 to 656 (Table 1).

**Table 1:** Patient characteristics in studies included in the meta-analysis.

Study reference, Year	Study name	Study years	Study location	Study phase	Blinding	Arterial occlusion required	Time to randomization; endovascular therapy	Allocation	Study population, n	Allocated therapy	Symptom onset to therapy time, h, median (IQR)	Age, mean (SD)	Baseline NIHSS median (range)
del Zoppo GJ, 1998[18]	PROACT	1994–1995	Canada, USA	2	Double-blind	Yes	within 6 hrs; within 6 hrs	Controls	14	heparin	5.7	69.6 (11.1)	19
								Cases	26	IA r-pro UK	5.4	66.5 (11.0)	17
Furlan A, 1999[1]	PROACT II	1996–1998	Canada, USA	3	Open design with blinded follow-up	Yes	NA; within 6 hrs	Controls	59	heparin	NA	64 (14)	17 (4–28)
								Cases	121	IA r-pro UK + heparin	5.3	64 (14)	17 (5–27)
Keris V, 2001[20]		1997–1998	Latvia	NA	Open-label	No	NA; within 6 hrs	Controls	33	heparin	NA	65 (8)	26 (5)^†^
								Cases	12	IA/IV tPA + heparin	4.0	53 (9)	25 (3) ^†^
Ducrocq X, 2005[19]		1995–1997	France	NA	Open design with blinded follow-up	No	NA; within 6 hrs	Controls	14	IV UK	4.1	58	14.6^†^
								Cases	13	IA UK	5.3	59.5	15.2^†^
Macleod MR, 2005[21]	Australian Urokinase Stroke Trial	1996–2003	Australia, New Zealand	NA	Open design with blinded follow-up	Yes	NA; within 24 hrs	Controls	8	heparin	12.5 (3.4–22.5)^§^	63.7 (12.3)	18 (5–29)
								Cases	8	IA UK + heparin	11.8 (5.8–21.8)^§^	64.2 (11.1)	23 (7–29)
Ogawa A, 2007[2]	MELT	2002–2005	Japan	NA	Open-label	Yes	within 6 hrs; within 6 hrs	Controls	57	heparin	NA	67.3 (8.5)	14 (6.8)^‡^
								Cases	57	IA UK + heparin	3.8^†^	66.9 (9.3)	14 (8.0)^‡^
Ciccone A, 2010[17]	SYNTHESIS pilot	2004–2008	Italy	NA	Open design with blinded follow-up	No	NA; within 6 hrs	Controls	29	IV tPA	2.6 (2.3–2.8)	64.0 (11.7)	16 (3–24)
								Cases	25	IA tPA	3.3 (2.8–4.0)	60.6 (13.7)	17 (2–26)
Broderick JP, 2013[8]	IMS III	2006–2012	Australia, Canada, Europe, USA	3	Open design with blinded follow-up	No	within 3 hrs 40 min; within 5 hrs	Controls	222	IV tPA	2.0 (0.6) ^†^	68 (23–84)*	16 (8–30)
								Cases	434	IV tPA + thrombectomy	2.0 (0.6) ^†^	69 (23–89)*	17 (7–40)
Ciccone A, 2013[9]	SYNTHESIS	2008–2012	Italy	NA	Open design with blinded follow-up	No	within 4.5 hrs; within 6 hrs	Controls	181	IV tPA	2.5 (2.2–3.2)	67 (11)	13 (3–24)
								Cases	181	IA tPA + thrombectomy	3.5 (3.1–4.2)	66 (11)	13 (2–26)
Kidwell CS, 2013[10]	MR RESCUE	2004–2011	Canada, USA	2	Open design with blinded follow-up	Yes	within 8 hrs; within 8 hrs	Controls	54	standard care	NA	67.1 (16.5)	17^†^
								Cases	64	IA tPA + thrombectomy		64.2 (12.8)	17^†^

NA = not available; IA = intra-arterial; IV = intravenous; r-pro UK = recombinant pro- urokinase; tPA = tissue plasminogen activator; h = hours; IQR = interquartile range;

* = median (range);

^†^ = mean (SD);

^‡^ = median (IQR);

^§^ = mean (range).

### The Cochrane risk of bias evaluation

Most of studies had low risk of bias in most of the assessed items (Table 2). One trial had high risk of bias in 4 items [20] and three trials had high risk of bias in 2 items [8,17,19]. Blinding of participants and personnel was the item with more studies having high risk of bias [8–10,17,19,20]; randomization sequence generation was unclear in four studies [17–19,21].

**Table 2:** Cochrane assessment of bias risk of randomized controlled trials.

	Del Zoppo GJ 1998[18]	Furlan A 1999[1]	Keris V 2001[20]	Ducrocq X 2005[19]	Macleod MR 2005[21]	Ogawa A 2007[2]	Ciccone A 2010[17]	Broderick JP 2013[8]	Ciccone A 2013[9]	Kidwell CS 2013[10]
Randomization sequence generation	Unclear	Low	High	Unclear	Unclear	Low	Unclear	Low	Low	Low
Allocation concealment	Low	Low	High	Low	Low	Low	Low	Low	Low	Low
Blinding of participants and personnel	Low	Low	High	High	Low	Low	High	High	High	High
Blinding of outcome assessment	Low	Low	High	High	Low	Low	Low	Low	Low	Low
Incomplete outcome data	Low	Low	Low	Low	Low	Low	High	Low	Low	Low
Selective outcome reporting	Low	Low	Low	Low	Low	Low	Low	Low	Low	Low
Other sources of bias	Low	Low	Low	Low	Low	Low	Unclear	High	Unclear	Low

### GRADE Quality of the Evidence

The quality of evidence for the effect of IA therapy on critical outcomes like mRS ≤2, mortality and sICH was low (Appendix B in S1 File).

### Meta-analysis of benefits and harms of endovascular therapy in ischemic stroke

**Fig 2:**
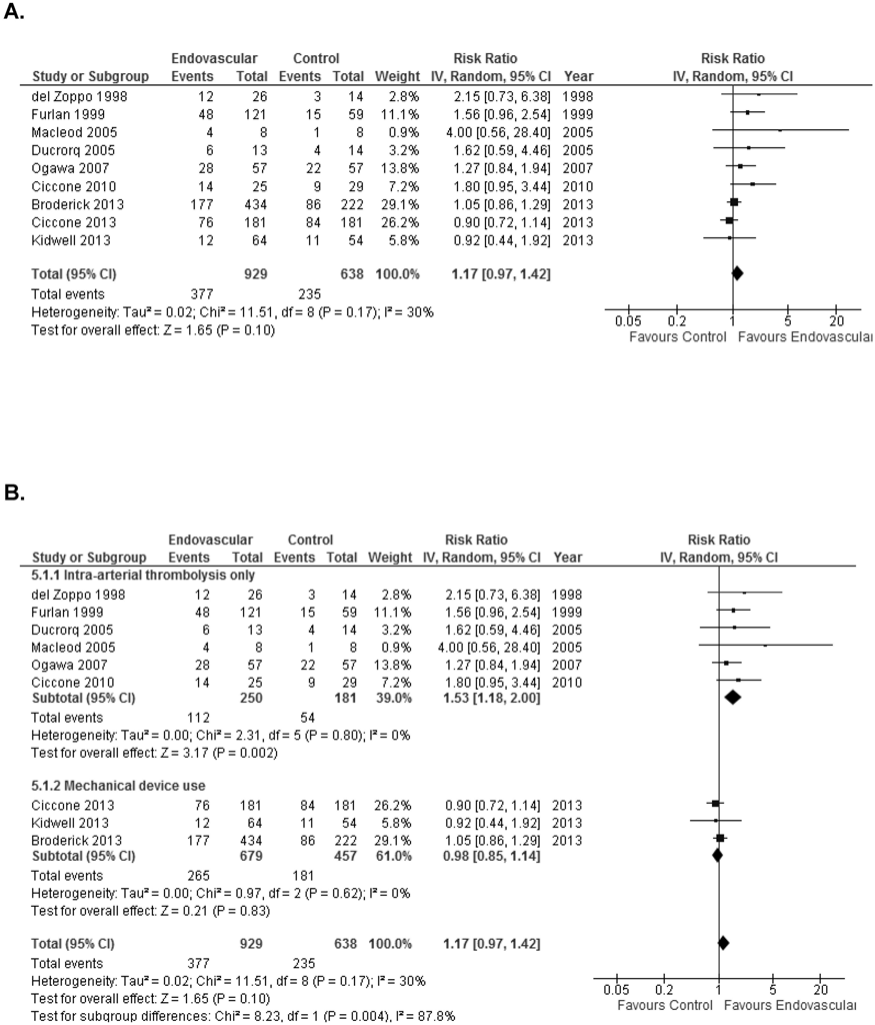
Forest plots showing modified Rankin Score 0–2 at 90 days between endovascular therapy and controls. **A:** All studies. **B:** Subgroup 1: IA thrombolysis only versus mechanical device use. **C:** Subgroup 2: Comparator includes IV thrombolysis versus no thrombolysis. **D:** Subgroup 3: Studies that required vessel occlusion versus studies did not require vessel occlusion status.

**Fig 3:**
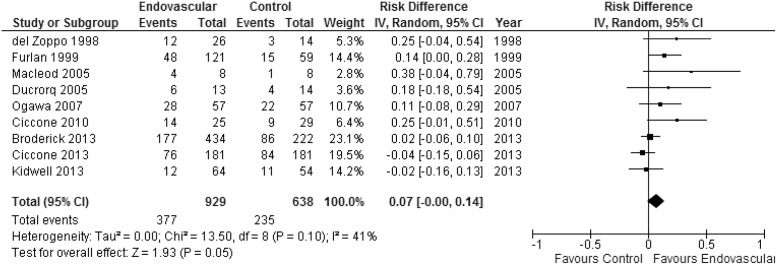
Modified Rankin Score 0–2 at 90 days expressed as absolute risk differences (ARD).

**Fig 4:**
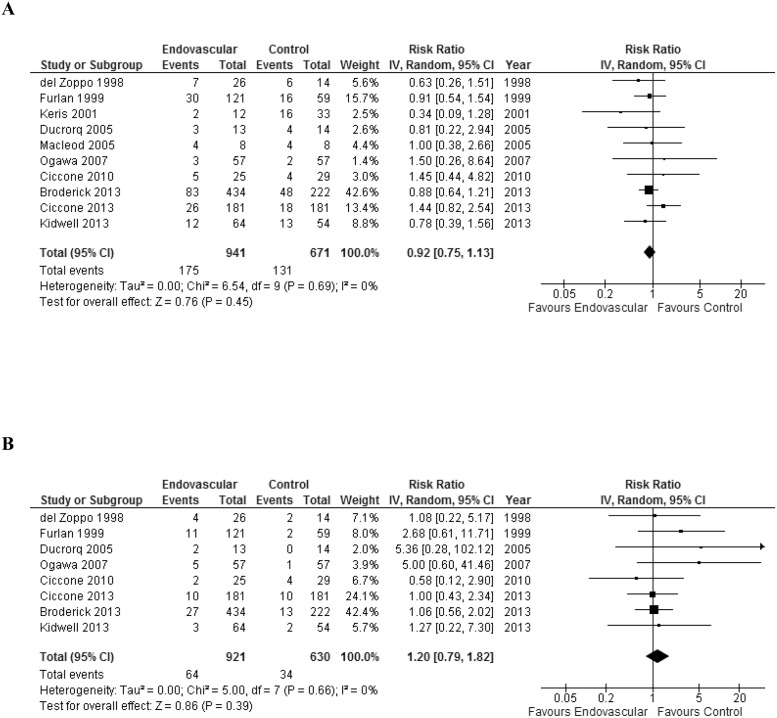
**A:** Forest plots showing mortality between endovascular therapy and controls. **B:** Forest plots showing symptomatic intracranial hemorrhage between endovascular therapy and controls.

We did not find a significant higher probability of beneficial outcome defined as mRS ≤2 with endovascular therapy in comparison to controls (RR = 1.17; 95% CI 0.97–1.42; p = 0.10). There was moderate heterogeneity among studies (I^2^ = 30%) (Fig 2A). Cumulative meta-analysis showed that the significant association between IA therapy and the beneficial primary outcome went towards zero and became non-significant over time (Fig A in S1 File). In absolute terms, there was no higher proportion of good outcomes (ARD 7%; 95% CI -0.1% to 14%; p = 0.05) (Fig 3). There were no significant difference in mortality when endovascular therapy was compared with control groups (RR = 0.92; 95% CI 0.75–1.13; p = 0.45) and no heterogeneity among studies (I^2^ = 0%) (Fig 4A). Endovascular therapy did not change the rate of sICH when compared with control group (RR = 1.20; 95% CI 0.79–1.82; p = 0.39) and no heterogeneity among studies (I^2^ = 0%) (Fig 4B). There was a significant higher probability of any ICH in controls in comparison to endovascular therapy group (RR = 1.47; 95% CI 1.14–1.90; p = 0.003; I^2^ = 45%) (Fig B in S1 File).

There was evidence of asymmetry of the funnel plots for the primary outcome, Rankin < 3 and mortality to suggest publication bias (p = 0.2, p = 0.2, respectively) (Fig F1-2 in S1 File). There was evidence of asymmetry of the funnel plots for the ICH outcomes to suggest publication bias (any ICH p = 0.03, symptomatic ICH p = 0.06) (Fig F3-4 in S1 File). For forest plots of absolute differences for the secondary outcomes (mortality and sICH) please see Fig G1-2 in S1 File.

### Subgroup analyses for the primary outcome

IA thrombolysis only was associated with higher chance of good outcome in terms of disability (RR = 1.53; 95% CI 1.18–2.00; p = 0.002) whereas mechanical device usage was not associated with increase in good outcome in terms of disability (Fig 2B). There were no significant differences between control group and endovascular group in the studies that required IV thrombolysis in the comparator. When comparator had no IV thrombolysis, the endovascular group showed a significant beneficial outcome than control group (RR = 1.38; 95% CI 1.04–1.82; p = 0.03) (Fig 2C). Analysis by vessel occlusion demonstrated that endovascular therapy was associated with the increased good outcome (RR = 1.38; 95% CI 1.04–1.82; p = 0.03) in the studies that required vessel occlusion for randomization. There were no significant difference between endovascular group and control group when vessel occlusion status was not required (Fig 2D). Heterogeneity ranged from 0% to 39% on subgroup analyses.

### Subgroup analyses for the secondary outcomes

In general there were no differences in subgroup analyses when compared with the main analyses. Forest plots of these subgroup analyses are shown in Fig C-E in S1 File.

## Discussion

In our study, ten RCTs were detected and 9 RCTs estimated good outcome defined as modified Rankin scale 2 or less. Endovascular therapy did not increase good outcome, and there was moderate heterogeneity. Similarly, symptomatic ICH and mortality in endovascular groups occur as frequently as those in control groups. Subgroup analyses showed that endovascular therapy increased beneficial outcome without heterogeneity if only IA thrombolysis was included in the active treatment group, if IV thrombolysis was not included in control groups or if subjects in studies required the evidence of vessel occlusion. The quality of evidence was low for all outcomes and the recommendation is weak for the use of IA therapy as per GRADE methodology.

Two prior systematic reviews only focused on the comparison between IA thrombolysis and controls.[11,12] The results of these studies are congruent with our subgroup analysis of endovascular therapy that used IA thrombolytics and demonstrated IA thrombolysis to reduce disability. Lee *et al*. showed that the patients treated with IA fibrinolysis were significantly more likely to have good clinical outcome defined as mRS 0–2 than conventional treatment without IV thrombolysis. (OR = 2.05; 95% CI 1.33–3.14; p = 0.001) [10]. Fields *et al*. also reported similar result for the patients with acute ischemic stroke due to MCA occlusion. (OR = 1.9; 95% CI 1.2–3.0).[11] Nam *et al*. reported a meta-analysis of RCTs comparing endovascular therapy to controls but included only a small number of patients who underwent mechanical thrombectomy.[13] This study included patients treated with IV thrombolysis in the control arms. The results showed that IA thrombolysis reduced poor outcome patients defined as mRS 3–6 compared with control treatments (RR = 0.80; 95% CI 0.67–0.95; p = 0.001), although IA thrombolysis did not have clear benefit over IV thrombolysis (RR = 0.68; 95% CI 0.46–1.00; p = 0.05). [13]

Our subgroup analyses indicated that IA thrombolysis compared to mechanical thrombectomy might be a factor for a study to demonstrate a benefit of endovascular approach. But arterial recanalization rates are lower in IA thrombolysis compared to mechanical thrombectomy and two of the three trials designated as allowing mechanical approach had majority of subjects undergo IA thrombolysis.[8,9,22] Comparing to IV thrombolysis may diminish the effect of IA therapy, especially if cohort includes those without target vessel occlusion or recanalized with IV thrombolysis.[8,9,17] We hypothesize that vessel occlusion is the most important factor. IV thrombolysis has lower recanalization rate and is less effective for recanalization of large vessel occlusion than IA therapy.[23,24] A sub study of IMS3 studied arterial occlusion pre- and post-treatment using CT angiography. Within the subgroup of patients with proximal large arterial occlusion at baseline, good mRS was observed more frequently in the endovascular treatment group than in the control group.[25] Including those without proximal arterial occlusion that are not amenable to endovascular approach dilutes the overall of effect of such approach towards the null. Several used NIHSS cut-offs which is highly correlated with vascular occlusion after IV t-PA ref. However, IMS3 had 19% of patients (80/423) who were randomized to IA arm. This is in accordance with NIHSS of 10 or greater having 70–80% specificity in having vascular occlusion. But 20% may be too high of a rate of including patients who would not qualify for the therapy. Future studies of endovascular therapy should enroll only those with target vessel occlusion.

We found mortality were similar to previous meta-analysis which reported there was no significant difference between endovascular treatment arms and control arms.[11,13] Our finding of no increase in sICH differs from previous reviews.[12] Our study included studies of mechanical thrombectomy and more studies with comparator arms including IV TPA, which is known to increase sICH. It is reported that IA thrombolysis caused more ICH than control treatment without IV thrombolysis;[12] however IA thrombolysis did not increase symptomatic hemorrhage compared with IV thrombolysis.

The quality of evidence of was assessed to be low by GRADE methodology. This may have several reasons. This methodology may have inherent limitations in evaluating this type of therapy and may result in low quality. The acute nature of stroke treatment prevents a double blind design with sham intervention. Risks of sedation or anesthesia should be a part of the endovascular approach and control sham procedure would not suffice. A prospective randomized open blinded endpoint (PROBE) design is the most realistic approach for clinical trials of acute endovascular therapy. The “imprecision” of the outcomes is somewhat inherent with scarce outcomes of ischemic stroke patients. In addition to heterogeneity of the outcomes of any strokes, inclusion of occlusions is another issue. However, the GRADE evaluation points to the need for more uniform approaches and more studies to increase precision of estimate of effects.

There were limitations in this study. First, study design, subject selection, and endovascular techniques varied among studies. For example we didn’t analyze time to treatment because these reviews referred to various time to treatment. Second, not all outcomes we evaluated were reported in the detected trials. Primary outcome defined mRS 0–2 were reported in 9 trials; Keris *et al*. regarded good outcome as mRS 3 or less and this outcomes was not analyzed ^20^. Finally, while mortality was described in all trials, not all trials described systematic imaging to detect any intracranial hemorrhage or reported symptomatic hemorrhages.

## Supporting Information

S1 File Appendix A: PubMed search Strategy. Appendix B: Grading Quality of the Evidence and Recommendations. Table A: PRISMA Checklist. Table B: GRADE evaluation of quality of evidence. Table C: List of excluded studies. Figure A: Cumulative meta-analysis (i.e. effects over time). Figure B: Forest plots showing any intracranial hemorrhage between endovascular therapy and controls. Figure C: Forest plot showing mortality in endovascular therapy vs controls. Figure C1: IA thrombolysis only vs mechanical device use. Figure C2: Comparator includes IV thrombolysis vs no thrombolysis. Figure C3: Studies that required vessel occlusion vs studies did not require vessel occlusion status. Figure D: Forest plot showing any intracranial hemorrhage in endovascular therapy vs controls. Figure D1: IA thrombolysis only vs mechanical device use. Figure D2: Comparator includes IV thrombolysis vs no thrombolysis. Figure D3: Studies that required vessel occlusion vs studies did not require vessel occlusion status. Figure E: Forest plot showing symptomatic intracranial hemorrhage in endovascular therapy vs controls. Figure E1: IA thrombolysis only vs mechanical device use. Figure E2: Comparator includes IV thrombolysis vs no thrombolysis. Figure E3: Studies that required vessel occlusion vs studies did not require vessel occlusion status. Figure F: Funnel Plots and Egger’s test for asymmetry of the funnel plot (p<0.1 for asymmetry). Figure F1: Primary outcome: Rankin <3. Egger’s test: p value = 0.2. Figure F2: Secondary outcome: Mortality. Egger’s test: p value = 0.2. Figure F3: Secondary outcome: Any ICH. Egger’s test: p value = 0.03. Figure F4: Secondary outcome: Symptomatic ICH. Egger’s test: p value = 0.06. Figure G: Absolute risk differences (ARD) for the secondary outcomes. Figure G1: Mortality. Figure G2: Symptomatic ICH.
(DOCX)
