# Supplementary material for: Acute Endovascular Reperfusion Therapy in Ischemic Stroke: A Systematic Review and Meta-Analysis of Randomized Controlled Trials
Source: PLoS One. 2015 Apr 27;10(4):e0122806. doi: 10.1371/journal.pone.0122806 (PMC4410940; doi:10.1371/journal.pone.0122806)
Supplement: S1 File — Appendix A: PubMed search Strategy. Appendix B: Grading Quality of the Evidence and Recommendations. Table A: PRISMA Checklist. Table B: GRADE evaluation of quality of evidence. Table C: List of excluded studies. Figure A: Cumulative meta-analysis (i.e. effects over time). Figure B: Forest plots showing any intracranial hemorrhage between endovascular therapy and controls. Figure C: Forest plot showing mortality in endovascular therapy vs controls. Figure C1: IA thrombolysis only vs mechanical device use. Figure C2: Comparator includes IV thrombolysis vs no thrombolysis. Figure C3: Studies that required vessel occlusion vs studies did not require vessel occlusion status. Figure D: Forest plot showing any intracranial hemorrhage in endovascular therapy vs controls. Figure D1: IA thrombolysis only vs mechanical device use. Figure D2: Comparator includes IV thrombolysis vs no thrombolysis. Figure D3: Studies that required vessel occlusion vs studies did not require vessel occlusion status. Figure E: Forest plot showing symptomatic intracranial hemorrhage in endovascular therapy vs controls. Figure E1: IA thrombolysis only vs mechanical device use. Figure E2: Comparator includes IV thrombolysis vs no thrombolysis. Figure E3: Studies that required vessel occlusion vs studies did not require vessel occlusion status. Figure F: Funnel Plots and Egger’s test for asymmetry of the funnel plot (p<0.1 for asymmetry). Figure F1: Primary outcome: Rankin <3. Egger’s test: p value = 0.2. Figure F2: Secondary outcome: Mortality. Egger’s test: p value = 0.2. Figure F3: Secondary outcome: Any ICH. Egger’s test: p value = 0.03. Figure F4: Secondary outcome: Symptomatic ICH. Egger’s test: p value = 0.06. Figure G: Absolute risk differences (ARD) for the secondary outcomes. Figure G1: Mortality. Figure G2: Symptomatic ICH. (DOCX) [file pone.0122806.s001.docx]

**Appendix A: PubMed search strategy**

((Acute[All Fields] AND ("ischemia"[MeSH Terms] OR "ischemia"[All Fields] OR "ischemic"[All Fields]) AND ("stroke"[MeSH Terms] OR "stroke"[All Fields])) OR (acute[All Fields] AND ("ischemia"[MeSH Terms] OR "ischemia"[All Fields] OR "ischaemic"[All Fields]) AND ("stroke"[MeSH Terms] OR "stroke"[All Fields])) OR (("ischemia"[MeSH Terms] OR "ischemia"[All Fields] OR "ischemic"[All Fields]) AND ("stroke"[MeSH Terms] OR "stroke"[All Fields])) OR (("ischemia"[MeSH Terms] OR "ischemia"[All Fields] OR "ischaemic"[All Fields]) AND ("stroke"[MeSH Terms] OR "stroke"[All Fields]))) AND (endovascular[All Fields] OR (endovascular[All Fields] AND ("therapy"[Subheading] OR "therapy"[All Fields] OR "therapeutics"[MeSH Terms] OR "therapeutics"[All Fields])) OR catheter-based[All Fields] OR intraarterial[All Fields] OR intra-arterial[All Fields] OR ("Intervention (Amstelveen)"[Journal] OR "Interv Sch Clin"[Journal]) OR Merci[All Fields] OR Penumbra[All Fields] OR Solitaire[All Fields] OR Trevo[All Fields] OR ("stents"[MeSH Terms] OR "stents"[All Fields] OR "stent"[All Fields]) OR (GpIIbIIIa[All Fields] AND antagonist[All Fields])) AND (("randomized controlled trial"[Publication Type] OR "randomized controlled trials as topic"[MeSH Terms] OR "randomized controlled trial"[All Fields] OR "randomised controlled trial"[All Fields]) OR ("randomized controlled trial"[Publication Type] OR "randomized controlled trials as topic"[MeSH Terms] OR "randomised controlled trial"[All Fields] OR "randomized controlled trial"[All Fields]) OR RCT[All Fields]) AND ("humans"[MeSH Terms] OR "humans"[All Fields])

**Appendix B: Grading Quality of the Evidence and Recommendations**

The quality of the evidence was assessed at the outcome level using the Grading of Recommendations Assessment, Development and Evaluation (GRADE) approach [[1](#_ENREF_1)]. This system specifies four categories for the quality of the evidence - high, moderate, low or very low-. These levels reflect the extent of our confidence in the estimates of an effect. Rating the quality of evidence begins with the design of the study. RCTs are considered high quality evidence but can be downgraded on the basis of five criteria: risk of bias, imprecision, inconsistency, indirectness, and publication bias.

**GRADE Quality of the Evidence**

The quality of evidence for the effect of IA therapy on critical outcomes like mRS ≤2, mortality and sICH was low (Supplementary Table 1). The quality of evidence for the effect on mRS≤2 at 90 days was downgraded two levels because of study limitations (risk of bias) and imprecision. The quality of evidence for mortality and sICH was rated down because of inconsistency and imprecision.

All of the included studies failed to achieve complete blinding. Eight out of ten studies reduced detection bias by blinding primary outcome assessors, but only two studies reduced even more this risk by blinding data analysts [[2](#_ENREF_2),[3](#_ENREF_3)]. Lack of blinding patients and health providers were deemed to affect the disability-free survival assessed with the mRS. Even though this outcome was blindly assessed, it was measured at 90 days on average with potential differences in interval treatment. In contrast, mortality and sICH were considered not to be affected in the same degree by the open label fashion design of the trials.

The quality of evidence was rated down for imprecision for all three outcomes. The CI of the pooled effect overlaps a RR of beneficial and harmful effects. Additionally, there was insufficient number of events needed for adequate power to detect a RR reduction at least ≥ 30% for sICH. We also downgraded inconsistency for mortality and sICH. The forest plot showed wide variations on both the magnitude and the direction of point estimates (beneficial and harmful effects).

Publication bias was minimized by a thorough search of databases without language and publication data restrictions. The risk of selective reporting bias was considered to be low in all studies as authors reported on outcomes listed in methods or the protocol was available. Although four studies were industry funded, the funding was disclosed or data management was performed independently of the sponsors. Additionally, finished but unpublished or ongoing studies were searched in the trials registration website.

**Table A: PRISMA Checklist**

| **Section/topic** | **#** | **Checklist item** | **Reported on page #** |
| --- | --- | --- | --- |
| **TITLE** | | |  |
| Title | 1 | Identify the report as a systematic review, meta-analysis, or both. | 1 |
| **ABSTRACT** | | |  |
| Structured summary | 2 | Provide a structured summary including, as applicable: background; objectives; data sources; study eligibility criteria, participants, and interventions; study appraisal and synthesis methods; results; limitations; conclusions and implications of key findings; systematic review registration number. | 2 |
| **INTRODUCTION** | | |  |
| Rationale | 3 | Describe the rationale for the review in the context of what is already known. | 3 |
| Objectives | 4 | Provide an explicit statement of questions being addressed with reference to participants, interventions, comparisons, outcomes, and study design (PICOS). | 3 |
| **METHODS** | | |  |
| Protocol and registration | 5 | Indicate if a review protocol exists, if and where it can be accessed (e.g., Web address), and, if available, provide registration information including registration number. | NA |
| Eligibility criteria | 6 | Specify study characteristics (e.g., PICOS, length of follow-up) and report characteristics (e.g., years considered, language, publication status) used as criteria for eligibility, giving rationale. | 4 |
| Information sources | 7 | Describe all information sources (e.g., databases with dates of coverage, contact with study authors to identify additional studies) in the search and date last searched. | 4 |
| Search | 8 | Present full electronic search strategy for at least one database, including any limits used, such that it could be repeated. | 4 |
| Study selection | 9 | State the process for selecting studies (i.e., screening, eligibility, included in systematic review, and, if applicable, included in the meta-analysis). | 4 |
| Data collection process | 10 | Describe method of data extraction from reports (e.g., piloted forms, independently, in duplicate) and any processes for obtaining and confirming data from investigators. | 4 |
| Data items | 11 | List and define all variables for which data were sought (e.g., PICOS, funding sources) and any assumptions and simplifications made. | 4 |
| Risk of bias in individual studies | 12 | Describe methods used for assessing risk of bias of individual studies (including specification of whether this was done at the study or outcome level), and how this information is to be used in any data synthesis. | 5 |
| Summary measures | 13 | State the principal summary measures (e.g., risk ratio, difference in means). | 5 |
| Synthesis of results | 14 | Describe the methods of handling data and combining results of studies, if done, including measures of consistency (e.g., I^2^) for each meta-analysis. | 5-6 |

| Risk of bias across studies | 15 | Specify any assessment of risk of bias that may affect the cumulative evidence (e.g., publication bias, selective reporting within studies). | 5 |
| --- | --- | --- | --- |
| Additional analyses | 16 | Describe methods of additional analyses (e.g., sensitivity or subgroup analyses, meta-regression), if done, indicating which were pre-specified. | 6 |
| **RESULTS** | | |  |
| Study selection | 17 | Give numbers of studies screened, assessed for eligibility, and included in the review, with reasons for exclusions at each stage, ideally with a flow diagram. | 7 |
| Study characteristics | 18 | For each study, present characteristics for which data were extracted (e.g., study size, PICOS, follow-up period) and provide the citations. | 7-8 |
| Risk of bias within studies | 19 | Present data on risk of bias of each study and, if available, any outcome level assessment (see item 12). | 11-12 |
| Results of individual studies | 20 | For all outcomes considered (benefits or harms), present, for each study: (a) simple summary data for each intervention group (b) effect estimates and confidence intervals, ideally with a forest plot. | 13 |
| Synthesis of results | 21 | Present results of each meta-analysis done, including confidence intervals and measures of consistency. | 13-14 |
| Risk of bias across studies | 22 | Present results of any assessment of risk of bias across studies (see Item 15). | 13 |
| Additional analysis | 23 | Give results of additional analyses, if done (e.g., sensitivity or subgroup analyses, meta-regression [see Item 16]). | 14 |
| **DISCUSSION** | | |  |
| Summary of evidence | 24 | Summarize the main findings including the strength of evidence for each main outcome; consider their relevance to key groups (e.g., healthcare providers, users, and policy makers). | 15 |
| Limitations | 25 | Discuss limitations at study and outcome level (e.g., risk of bias), and at review-level (e.g., incomplete retrieval of identified research, reporting bias). | 17 |
| Conclusions | 26 | Provide a general interpretation of the results in the context of other evidence, and implications for future research. | 18 |
| **FUNDING** | | |  |
| Funding | 27 | Describe sources of funding for the systematic review and other support (e.g., supply of data); role of funders for the systematic review. | 19 |

*From:*  Moher D, Liberati A, Tetzlaff J, Altman DG, The PRISMA Group (2009). Preferred Reporting Items for Systematic Reviews and Meta-Analyses: The PRISMA Statement. PLoS Med 6(6): e1000097. doi:10.1371/journal.pmed1000097

For more information, visit: **www.prisma-statement.org**.

**Table B: GRADE evaluation of quality of evidence**

| **Patient or population:** patients with Acute ischemic stroke  **Settings:** tertiary care hospitals **Intervention:** Endovascular treatment (thrombolytic or mechanical reperfusion) **Comparison:** other (intravascular thrombolytic, anticoagulation or placebo) | | | | | | |
| --- | --- | --- | --- | --- | --- | --- |
| **Outcomes** | **Illustrative comparative risks* (95% CI)** | | **Relative effect (95% CI)** | **No of Participants (studies)** | **Quality of the evidence (GRADE)** | **Comments** |
|  | Assumed risk | Corresponding risk |  |  |  |  |
|  | **Other (intravascular thrombolytic, anticoagulation or placebo)** | **Endovascular treatment (thrombolytic or mechanical reperfusion)** |  |  |  |  |
| **Rankin score 0-2**  Follow-up: 3-6 months | **372 per 1000** | **435 per 1000** (361 to 528) | **RR 1.17**  (0.97 to 1.42) | 1527 (9 studies) | ⊕⊕⊝⊝ **low**^1,2^ |  |
| **Mortality** | **195 per 1000** | **180 per 1000** (146 to 221) | **RR 0.92**  (0.75 to 1.13) | 1612 (10 studies) | ⊕⊕⊝⊝ **low**^2,3^ |  |
| **Symptomatic intracranial hemorrhage** | **54 per 1000** | **65 per 1000** (43 to 98) | **RR 1.20**  (0.79 to 1.82) | 1551 (8 studies) | ⊕⊕⊝⊝ **low**^3,4^ |  |
| *The basis for the **assumed risk** (e.g. the median control group risk across studies) is provided in footnotes. The **corresponding risk** (and its 95% confidence interval) is based on the assumed risk in the comparison group and the **relative effect** of the intervention (and its 95% CI).  **CI:** Confidence interval; **RR:** Risk ratio; | | | | | | |
| GRADE Working Group grades of evidence **High quality:** Further research is very unlikely to change our confidence in the estimate of effect.  **Moderate quality:** Further research is likely to have an important impact on our confidence in the estimate of effect and may change the estimate. **Low quality:** Further research is very likely to have an important impact on our confidence in the estimate of effect and is likely to change the estimate. **Very low quality:** We are very uncertain about the estimate. | | | | | | |
| ^1^ Open label trials (patients and health care providers unblinded). Although seven studies blinded outcome assessors, only two reduced detection bias by blinding data analysts too. Five out of eight studies with unclear description of sequence generation. There was high risk of attrition bias for one study and one study with unclear allocation concealment.  ^2^ The CI of the pooled effect overlaps a relative risk of harmful and beneficial effects.  ^3^ Wide variations in point estimates with some studies suggesting benefit and others harm  ^4^ There were fewer than 300 events in total in the intervention and control groups. Insufficient number of events needed for adequate power to detect a relative risk reduction ≥ 30% | | | | | | |

**Table C: List of excluded studies**

|  | First Author | Year published | PMID | Study details | Reason for exclusion |
| --- | --- | --- | --- | --- | --- |
| 1 | Kase CS | 2001 | 11706099 | PROACT II trial subjects; analysis of frequency, clinical characteristics, and predictors of symptomatic ICH | subgroup analysis of an included study |
| 2 | Lang W | 1997 | 9420947 | Title: Thrombolysis in acute ischemic stroke | Review paper |
| 3 | Lewandowski CA | 1999 | 10582984 | Combined Intravenous and Intra-Arterial r-TPA Versus Placebo + Intra-Arterial Therapy of Acute Ischemic Stroke | Endovascular therapy in both groups |
| 4 | Nogueira RG | 2012 | 22932714 | Trevo versus Merci retrievers for thrombectomy revascularisation of large vessel occlusions in acute ischaemic stroke | Endovascular therapy in both groups |
| 5 | Popiela TJ | 2010 | 20687361 | intravenous (n=4) vs intra-arterial thrmobolysis (n=2) | n too small |
| 6 | Rahme R | 2013 | 23223507 | PROACT II trial subjects subgroup analysis; Is Intra-Arterial Thrombolysis Beneficial for M2 Occlusions? | subgroup analysis of an included study |
| 7 | Saver JL | 2012 | 22932715 | Solitaire flow restoration device versus the Merci Retriever in patients with acute ischaemic stroke | Endovascular therapy in both groups |
| 8 | Sen S | 2009 | 19277904 | IV (n=4) vs IA TPA (n=3) in Acute Ischemic Stroke with CT Evidence of Major Vessel Occlusion: A Feasibility Study | n too small |
| 9 | Wang F | 2007 | Not PubMed indexed | IV + IA TPA thrombolysis group (n = 60) vs IA TPA thrombolysis group (n = 46) | Endovascular therapy in both groups |
| 10 | Wolfe T | 2008 | 18436152 | Comparison of Combined Venous and Arterial Thrombolysis with Primary Arterial Therapy Using Recombinant t-PA in Acute Ischemic Stroke | Endovascular therapy in both groups |

**Figure A:** Cumulative meta-analysis (i.e. effects over time): Cumulative meta-analysis shows that the significant association between IA therapy and the beneficial primary outcome went towards zero and became non-significant over time.

**Figure B:** Forest plots showing any intracranial hemorrhage between endovascular therapy and controls.


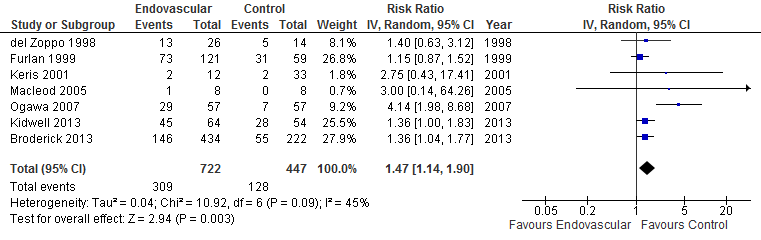


**Figure C:** Forest plot showing mortality in endovascular therapy vs controls

**Figure C1:** IA thrombolysis only vs mechanical device use


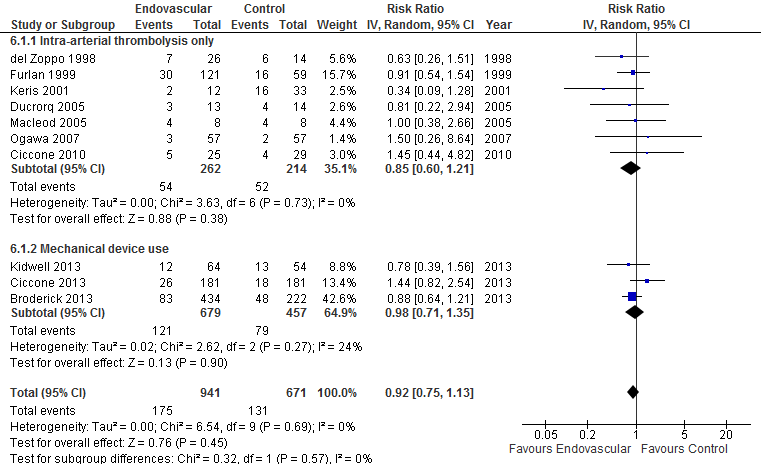


**Figure C2:** Comparator includes IV thrombolysis vs no thrombolysis


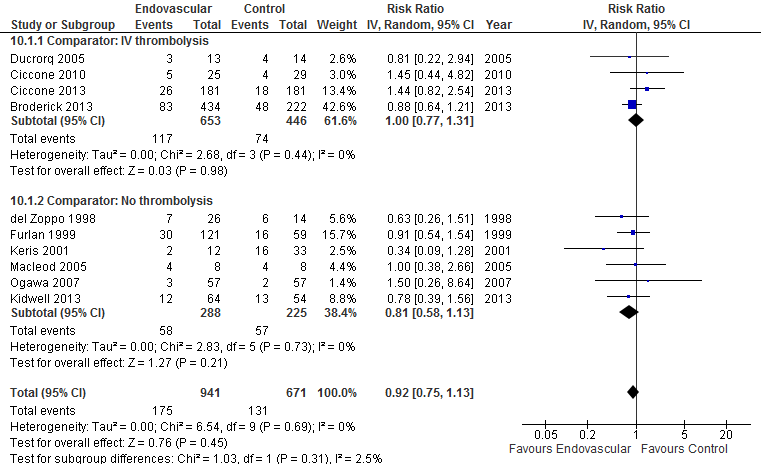


**Figure C3:** Studies that required vessel occlusion vs studies did not require vessel occlusion status


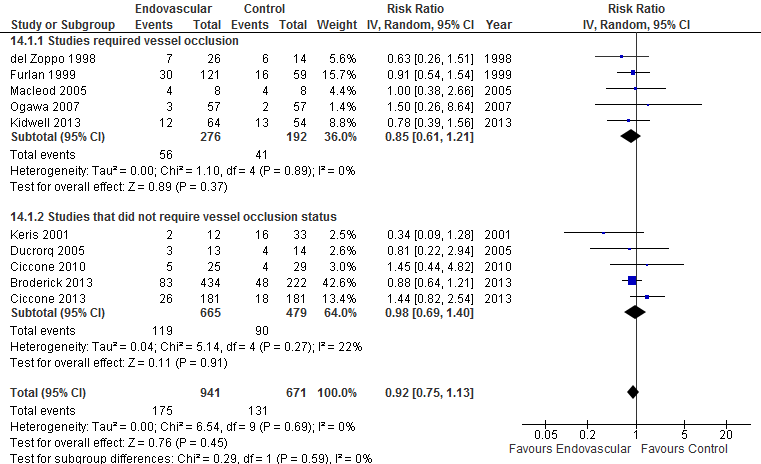


**Figure D:** Forest plot showing any intracranial hemorrhage in endovascular therapy vs controls

**Figure D1:** IA thrombolysis only vs mechanical device use


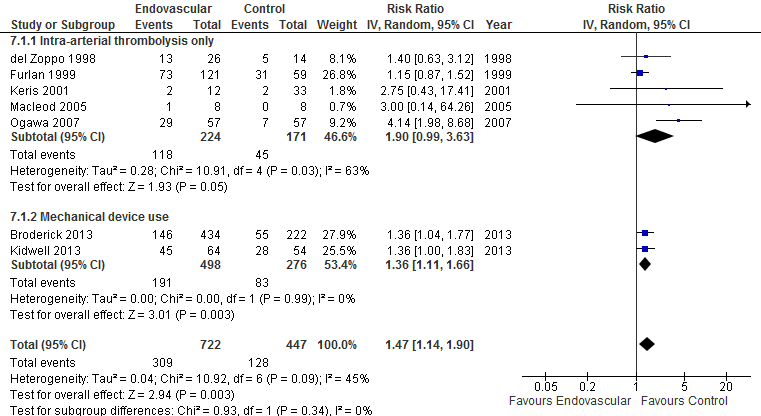


**Figure D2:** Comparator includes IV thrombolysis vs no thrombolysis


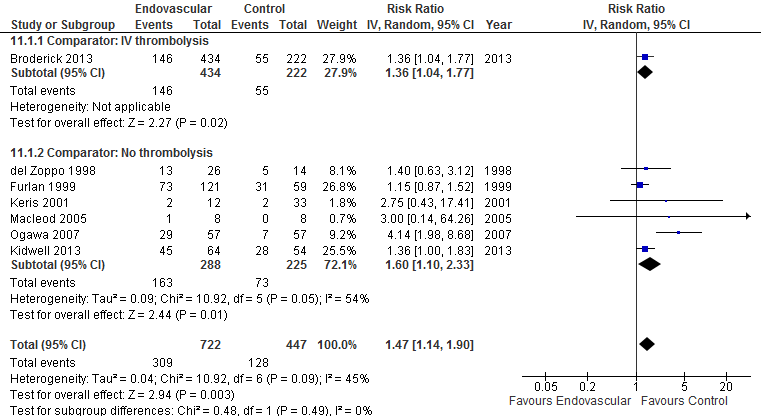


**Figure D3:** Studies that required vessel occlusion vs studies did not require vessel occlusion status


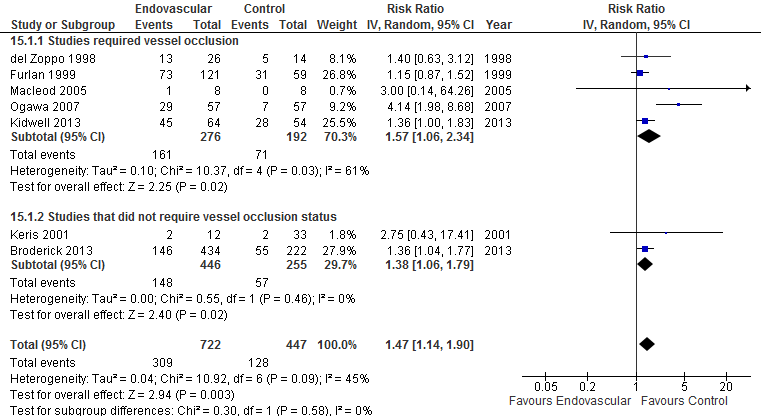


**Figure E:** Forest plot showing symptomatic intracranial hemorrhage in endovascular therapy vs controls

**Figure E1:** IA thrombolysis only vs mechanical device use


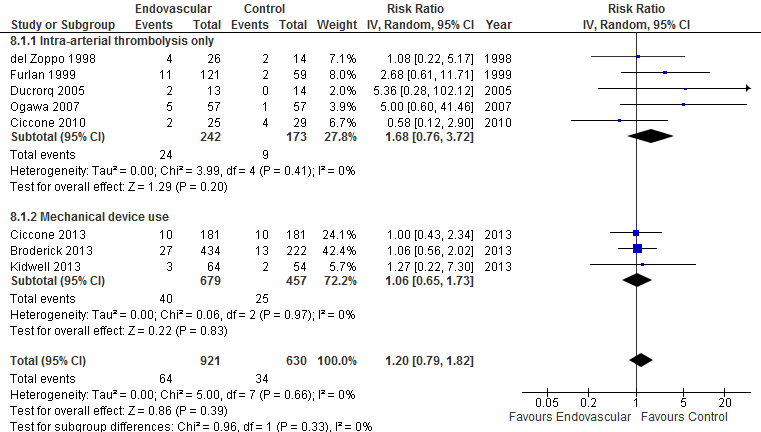


**Figure E2:** Comparator includes IV thrombolysis vs no thrombolysis


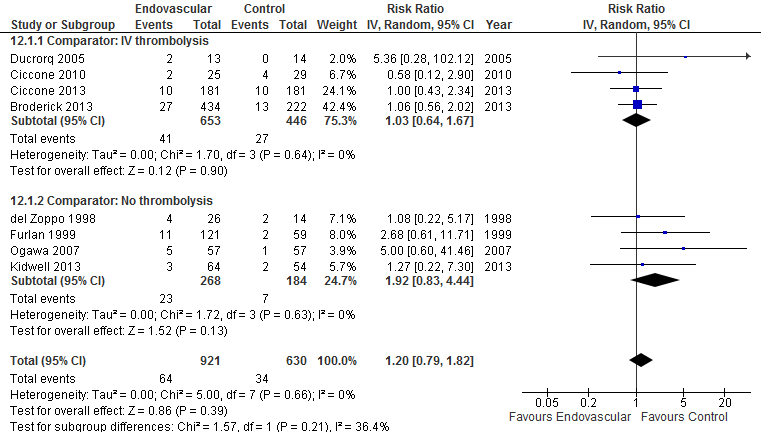


**Figure E3:** Studies that required vessel occlusion vs studies did not require vessel occlusion status


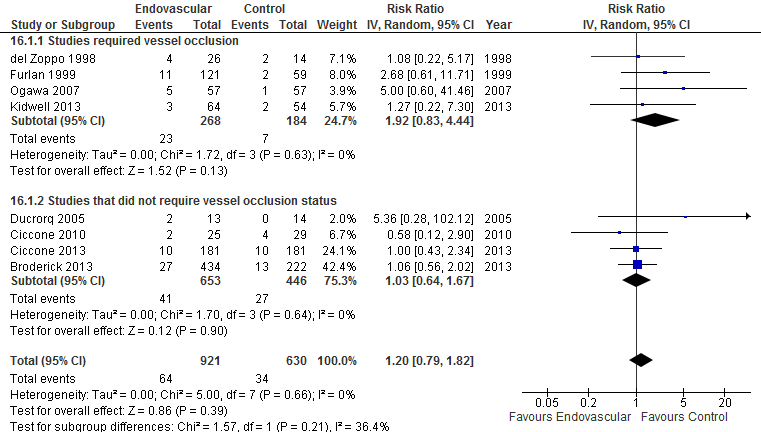


**Figure F:** Funnel Plots and Egger’s test for asymmetry of the funnel plot (p<0.1 for asymmetry)

**Figure F1:**  Primary outcome: Rankin <3. Egger’s test: p value=0.2


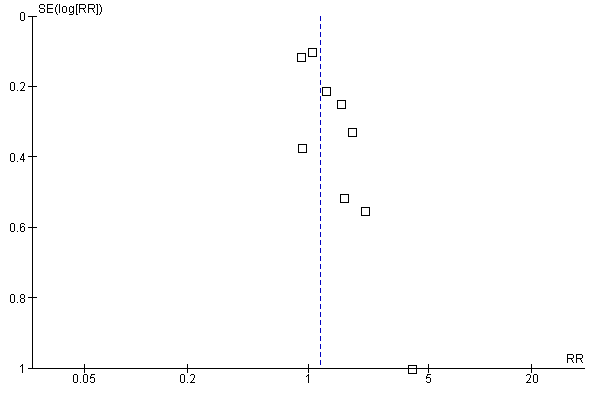


**Figure F2:** Secondary outcome: Mortality. Egger’s test: p value=0.2


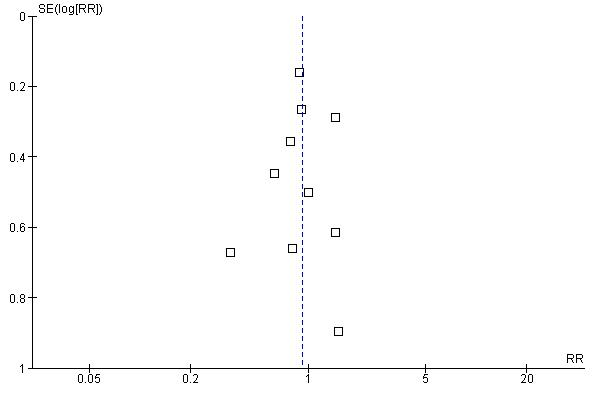


**Figure F3:** Secondary outcome: Any ICH. Egger’s test: p value=0.03


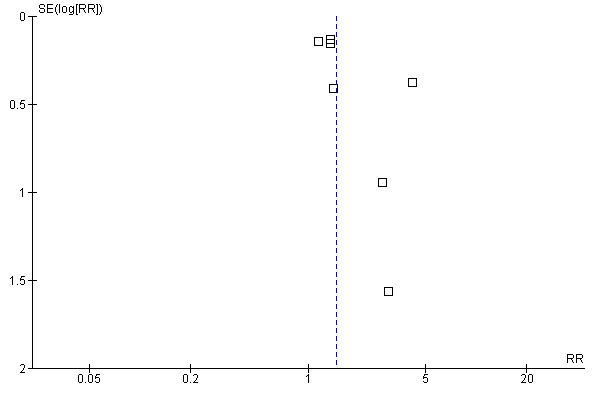


**Figure F4:** Secondary outcome: Symptomatic ICH. Egger’s test: p value=0.06


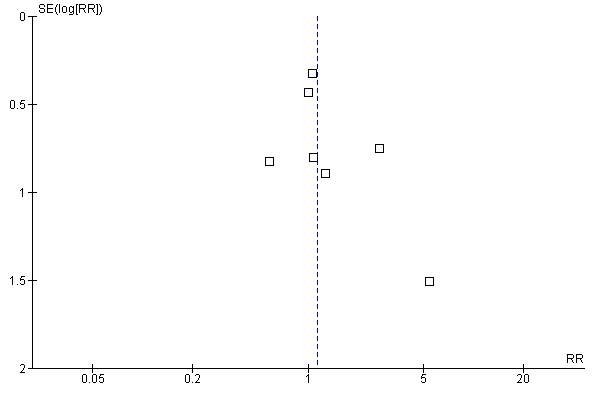


**Figure G:** Absolute risk differences (ARD) for the secondary outcomes

**Figure G1:** Mortality


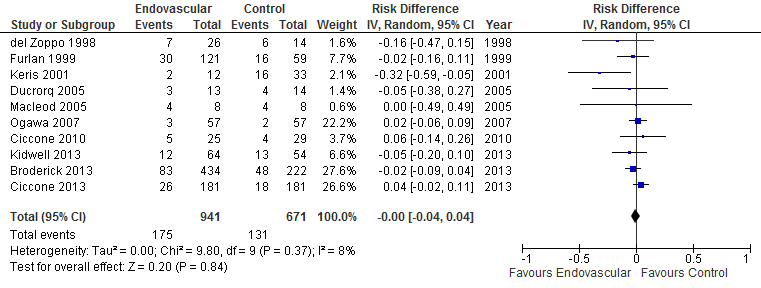


**Figure G2:** Symptomatic ICH


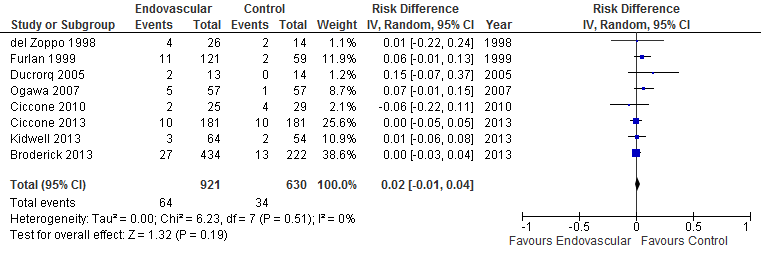


References:

1. Guyatt GH, Oxman AD, Vist GE, Kunz R, Falck-Ytter Y, et al. (2008) GRADE: an emerging consensus on rating quality of evidence and strength of recommendations. BMJ 336: 924-926.

2. Ciccone A, Valvassori L, Ponzio M, Ballabio E, Gasparotti R, et al. (2010) Intra-arterial or intravenous thrombolysis for acute ischemic stroke? The SYNTHESIS pilot trial. J Neurointerv Surg 2: 74-79.

3. Ciccone A, Valvassori L, Nichelatti M, Sgoifo A, Ponzio M, et al. (2013) Endovascular treatment for acute ischemic stroke. N Engl J Med 368: 904-913.
